# Confounding Factors and Their Mitigation in Measurements of Bioelectrical Impedance at the Skin Interface

**DOI:** 10.3390/bioengineering12090926

**Published:** 2025-08-28

**Authors:** Adrian Iftime, Cristian Scheau, Ramona-Madalina Babeș, Diana Ionescu, Argyrios Periferakis, Octavian Călinescu

**Affiliations:** 1 Department of Biophysics, The “Carol Davila” University of Medicine and Pharmacy, 8 Eroii Sanitari Bvd., 050474 Bucharest, Romania; ramona.babes@umfcd.ro (R.-M.B.); diana.ionescu@umfcd.ro (D.I.); octavian.calinescu@umfcd.ro (O.C.); 2 Department of Physiology, The “Carol Davila” University of Medicine and Pharmacy, 050474 Bucharest, Romania; cristian.scheau@umfcd.ro (C.S.);; 3 Department of Radiology and Medical Imaging, “Foisor” Clinical Hospital of Orthopaedics, Traumatology and Osteoarticular TB, 021382 Bucharest, Romania; 4 Akadimia of Ancient Greek and Traditional Chinese Medicine, 16675 Athens, Greece; 5 Elkyda, Research & Education Centre of Charismatheia, 17675 Athens, Greece

**Keywords:** bioelectrical impedance, measurement errors, skin–electrode interface, electric impedance spectroscopy, electric impedance tomography, bioelectric impedance analysis, bias

## Abstract

Background: Bioelectrical impedance measurement is a technique used in engineering (development of different wearable biosensors with diverse applications), in the medical field (health monitoring and diagnosis), and in biomedical research (fundamental and applied). Problem: despite its long history and existence of standardized techniques, measurement results can often be unreliable and plagued by high variance (intra- and inter-subject measurement), which can distort the correct interpretation of the results. Methods: we have reviewed bioelectrical impedance measurements from both engineering and medical research teams over the past 60 years, with a focus on factors that might influence the skin–electrode interface. Results: We identified 40 confounding factors in 10 different categories, some of which are sometimes overlooked in applied research, and we propose mitigation strategies for each one.

## 1. Overview of Physical Principles of Bioelectrical Impedance Techniques

### 1.1. Introduction

In the medical field, there is an ongoing need for faster, less invasive diagnostic and screening methods. For instance, for monitoring the evolution of a patient, standard magnetic resonance imaging (MRI) offers excellent spatial resolution, but the technique is very slow (takes tens of minutes to perform the data acquisition). X-ray computed tomography (CT) has a better temporal resolution (in the order of seconds) but has the downside of exposing the patient to ionizing radiation, imposing strict limits on the safe number of investigations that can be performed on a patient [1]. Both techniques require bulky equipment that cannot be used at the bedside. Consequently, there is a continuous research endeavor to find accurate, objective, easy-to-perform methods that can be used for diagnosing, characterizing illnesses, monitoring the internal physiological or pathological state, or for adjusting treatment.

Among these, bioelectrical impedance recordings of various tissues, organs, and body regions were highlighted as possible proxy indicators of “normal”, “abnormal”, or “different from average” for various anatomical or physiological conditions [2]. Electrical investigation methods for various medical needs have been developed over the past century, in research efforts that span multiple fields: medicine, biology, electrical engineering, physics, biophysics, chemistry, mathematics, and computer science.

In this study, we have reviewed research done on electrical impedance measurement applications: electrical impedance spectroscopy (EIS), electrical impedance tomography (EIT), and related various bioimpedance analysis techniques, with a focus on the research of the meeting point between the end of the measuring device (the electrode) and the living human (the skin). Impedance measurements began around 1920 (Gildemeister & Kaufhold, cit. in [3]) and are still plagued by both systematic and random errors, recognized from the early history of these measurements [4]. We reviewed (i) the problems in getting reliable measurements at the skin interface in the context of newer technologies (EIS/EIT) and (ii) the practical recommendations to deal with them (Section 2.1, Section 2.2, Section 2.3, Section 2.4, Section 2.5, Section 2.6, Section 2.7, Section 2.8, Section 2.9 and Section 2.10). A very short graphical overview of the EIS/EIT methods is provided in Section 1.2, as these techniques are presented in great detail in many published dedicated reviews [2,5,6,7,8,9,10,11,12,13].

### 1.2. Basic Principles of Bioelectrical Impedance Techniques

Electric current flows at different rates through complex structures such as living organisms. The general idea is that by careful analysis of the electrical parameters, one can infer a useful number of details about the structure and properties of living tissues.

#### 1.2.1. Electric Impedance Spectroscopy (EIS)

EIS is a technique that records and compares the electrical response of a system to a time varying electrical signal (usually AC current) [14,15]. It is a non-destructive method used to characterize the electrical properties of many systems [16,17] and provides structural or functional details of the respective systems [5,13,18].

**Figure 1:**
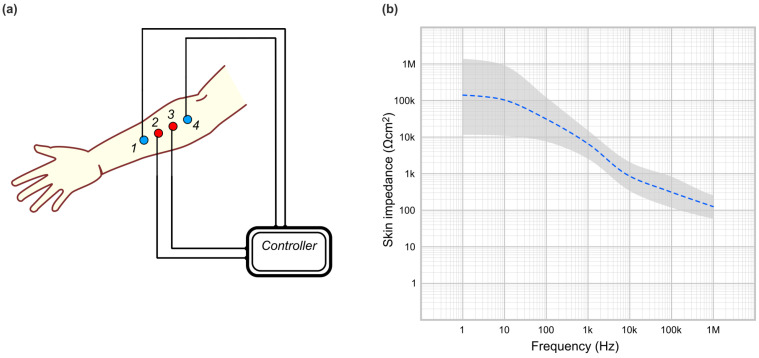
Electric impedance spectroscopy (EIS) overview. (**a**) Diagram of a typical four-electrode EIS technique. Current injection electrodes (1, 4) are marked with blue; recording electrodes (2, 3) are marked with red. (**b**) A typical impedance spectroscopy plot values of human skin (dashed blue line); shaded gray bands: intra- and inter-subject variation (from collating publicly available data from [21,22]).

EIS uses few electrodes, for instance, four (in four-electrode technique); see Figure 1a. Other methods exist (two-electrode method, three electrode method), but the presentation of these is beyond the purpose of this paper; detailed comparisons between these methods, used in general medical research, are available [7,16,19]. The four-electrode method was developed specifically to avoid errors that arise from electrode–solution interfaces [20]. The electrodes can be attached adjacent to each other on a small area of the skin (if the focus is the measurement of the impedance of the skin). The two outer electrodes are providing a path for an injected current. The two inner electrodes are recording electrodes, connected to a highly sensitive amplifier; they pick up the voltage differences created by the flow of the current induced by the outer electrodes. Electrodes can also be placed farther apart; for instance, in bioelectrical impedance analysis (BIA) two electrodes are attached on the arm and two on the leg, allowing the investigation of the current flow through all body tissues in between these locations. In this technique, the most distal two electrodes are current–injecting electrodes. The frequency of the alternating current can be varied, sweeping from a minimum to a maximum range depending on the application.

The results can be plotted graphically in various ways. The simplest is an impedance spectroscopy plot, where the recorded impedance values (vertical axis) are plotted against the frequency of the injected current (horizontal axis). See Figure 1b for a sample generic plot of the average impedance values of the human healthy skin (obtained by collating publicly available data from [21,22] analyzed with [23]); to ensure consistency, the validity was cross-referenced with previously published data from healthy subjects [4,19,24]. Typically, the human skin has 500 kΩ for 1 cm^2^ at 1Hz signal, but with huge intra- and inter-subject variation from 10 kΩ to 1 MΩ; the values have a non-linear decrease with increasing frequency [25]. The plotted shapes are typically downward curves, and with higher resolution (smaller frequency steps), certain “dips” can be observed at different frequency ranges, reflecting different structural properties; other electrical parameters can be plotted or derived from the raw impedance measurements using different electrical models of the skin [16,26,27,28].

#### 1.2.2. Electric Impedance Tomography (EIT)

EIT uses multiple electrodes attached to the skin, surrounding the target’s internal structures (heart, lungs, liver, etc.). Most EIT systems use 8 to 32 electrodes [29], and experimental 3D EIT uses up to 128 electrodes currently [30]; the resolution increases with the number of electrodes but at the cost of increasing the measurement time and thus limiting the imaging speed [10].

**Figure 2:**
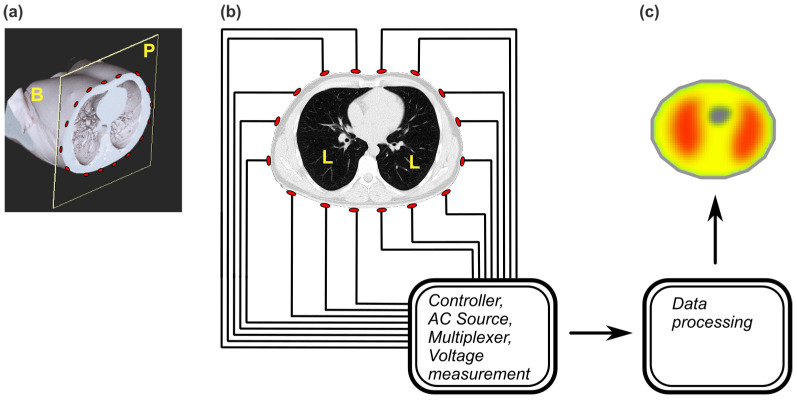
Electric impedance tomography (EIT) overview. (**a**) An example of placement of 16 electrodes (red ellipses) on a horizontal cross-section of the thorax on a section plane “P” through the body “B”. (**b**) The section plane intercepts the target organs (lungs “L” in this example). Electrodes (red ellipses) are connected with the EIT hardware. (**c**) An EIT “image” is a reconstructed map of the calculated distribution impedance changes in space and/or time.

Electrodes are usually placed at the intersection of the skin with a virtual section plane (P) through the body (Figure 2a); the plane contains the target organs (lungs in the thorax in this example). The electrodes are distributed in the section plane (Figure 2b). Electrodes are connected to a multiplexer and controller, which inject current to a pair of electrodes and record impedance values on the other electrodes. After a measurement, the controller selects another pair of electrodes as injecting and the rest as recording, and the cycle then repeats. Many variations exist in pairing (which pair of electrodes is activated), ordering and timing of activation, and number and types of electrodes [31]. A substantial effort is dedicated to the improvement of the hardware components that make possible such complex data acquisition in real time [10,32].

An EIT “image” (Figure 2c) is produced after a complex signal analysis that takes boundary measurements (at the skin level) as input parameters and reconstructs the most likely distribution of the internal electrical parameters that can produce the boundary values. It is a rather functional image (a distribution of changes of impedance in space and/or time). Although it is displayed as a section, it is not a true slice (like a computer tomography slice), but an EIT “sensitivity region” [33,34], essentially a map of reconstructed electrical parameters. These parameters depend primarily on the true impedance values of the structures in the plane of electrodes but are heavily influenced by the impedance values of the layers above and below [33]. The reconstruction methodologies and algorithms are the major breakthroughs in this technique; there are many variations, each with advantages and disadvantages [10,12,35]. The key point is the excellent temporal resolution (in the order of 50 acquisitions per second) [36], which makes EIT one of the best candidates for real-time clinical monitoring of pulmonary ventilation, pulmonary perfusion, cardiac output, etc. [8,34]. The main disadvantage is the low spatial resolution and the high sensitivity of the technique to measurement errors at the electrode–skin interface. A 5% error of the electrode–skin impedance was found to significantly distort EIT images, therefore making EIT application problematic in wider clinical situations [37]. The reduction of the error rates at the electrode–skin interface is considered as paramount to increase the EIT viability in clinical settings; many research projects are seeking to improve the electrodes and their reliability.

### 1.3. General Considerations About the Skin

In the above discussed techniques, the electrodes are attached to the skin. Regardless of the technique (BIA, EIS, EIT), the impedance of the skin is a constituent part of the result, and we briefly summarize the research on the structures that influence the electrical properties of the skin, highlighting the topics relevant for our discussion.

Human skin is a complex organ, with a layered structure (from outside to the inside): (i) stratum corneum (SC), (ii) viable epidermis, and (iii) dermis; these layers rest on (iv) fatty subcutaneous tissue (hypodermis) that contains larger blood vessels, nerves, and fibrous bands anchoring the skin to the deeper structures [38]. The first three layers have variable thickness and are penetrated by appendages (hair follicles and sweat glands). On top of the SC there is a variable amount of sebum (an oily like substance that acts as a dielectric). This layered structure is part of many electrical models of the human skin [19,39] and was recognized from early studies as one of the explanations for the variability of the results [4,24,25,40], the most variable component being the SC [7,40].

The hair follicles might have the hair inside (active follicle) or not (dormant or dead follicle). Associated with the follicles are the sebaceous glands that secrete the sebum that reaches the surface of the skin; hair follicles can have a tiny muscle attached (arrector pili), with a controlling nerve fiber. The contraction of this muscle squeezes out the sebum from sebaceous glands [41]; vigorous, generalized contraction of these muscles creates “goose bumps” (the pilomotor reflex). The base of the hair follicle has a degree of permeability [42], and as a whole it can be a conductive pathway (depending on hair position, follicle state).

The sweat glands are broadly classified into two types by the type of secretion and the location of the opening pore: (a) eccrine glands have the pore on the skin surface, and (b) apocrine glands, in or around the hair follicle opening. The opening (of both types) is at the end of the tube (duct) that carries the sweat produced at its other (deeper) end, in a tightly coiled portion, called the secretory base (secretory coil). The secretory coil is deeper in the dermis and sometimes reaches the hypodermis, thus creating a penetrating pathway through the skin. The properties and amount of sweat secreted are controlled by multiple mechanisms (by the nervous system and endocrine system), and the sweat is expelled to the surface through contractions of special cells (myoepithelial cells). Sweat is an ionic conductor and has multiple effects: it dramatically increases the conductivity inside the sweat gland, it moistens the SC and changes its electrical characteristics [7], and it spreads on the surface of the skin, equalizing skin impedance over an appreciable area [43].

To sum up, the sweat glands (and moistened hair follicles) are more likely to be preferred paths for electrical currents [44], but their permeability depends mainly on the activity of the sweat glands. In general, the conductance increases in hydrated skin, while the impedance changes in the opposite direction [45].

The main purposes of sweating are thermoregulation [46] and rapid flight-or-fight stress responses [47,48]. The activity of sweat glands is influenced by local conditions in the skin, and it is also under extensive neuronal control [49]. Therefore, any condition that might interfere with the metabolic changes that can alter thermoregulation, or any condition that might be potentially stressful, can induce a sweat response. These factors should be accounted for in BIA/EIS/EIT studies with electrodes placed on the skin, since these can induce or block sweating responses, acting as confounding factors.

The dermis layer, with its sweat glands, controls not only cooling via sweating but also controls the loss or retention of body heat through the numerous controlled arterioles (small arteries) that can regulate blood flow to the superficial capillary bed [38]. These arterioles dilate to fill up the capillary bed (vasodilation) with warm blood, therefore radiating heat or, conversely, can constrict to restrict blood flow and prevent heat loss. In addition to local thermal control [50], these mechanisms are also employed by the body to regulate blood pressure and flight-or-fight stress responses [51] and are under extensive nervous control [52]. It is known that an increase in blood flow in the skin leads to a reduction of skin impedance [53]. Therefore, any factor that can induce vasodilation or vasoconstriction can act as a confounding factor and potentially modify the impedance values read with skin electrodes.

In Section 2, we review the most common relevant clinical factors that can modify the skin physiology (triggering a sweat response, vasodilation, vasoconstriction, etc.) and a number of other structural or incidental factors that can also alter electrical recordings.

### 1.4. Challenges in Bioelectrical Impedance Measurements

There are two arms of this interdisciplinary research: (i) fundamental research aimed at understanding phenomena (mainly biophysics, medical physics, mathematical modeling) and (ii) applied research (bioengineering of sensors, development of computer algorithms, medical devices and various commercial consumer-oriented devices). Scientific research related to impedance technology was estimated at more than 1500 publications per year in this decade [54]. The amount and diversity of subdomains covered highlights the difficulties for researchers aiming at an overview of the field. From our teaching experience we have identified two main issues:

*Difficulties for medical researchers*: A huge corpus of knowledge has been accumulated during the past 50 years in what is called bio-electromagnetism, the field of biophysics that deals with the interaction of living tissue with electrical and magnetic phenomena. A key number of discovered processes can only be modeled and properly understood within the general framework of electromagnetism and fluid dynamics; these processes are often non-linear and/or have parameters that can only be described with advanced mathematics (for instance, complex numbers, vector spaces, finite element methods). To simplify, we do not have *words* in the usual day-to-day vocabulary to explicitly describe these phenomena; physicists had to coin precise terms that are hard to visualize (for instance, “lag of phase angle” or “complex space plane”). All these issues are high entry barriers for medical doctors or biologists that wish to include bioelectrical measurements in their research. Often, some engineering subtleties are therefore left ignored by medical doctors.

*Difficulties for engineers:* On the other hand, the engineers or physicists wishing to apply measuring-and-modeling methods to a living human are quickly confronted with the harsh reality that living tissues are of bewildering complexity and diversity. For instance, skin differs from anatomical site to anatomical site (in the same person), differs from person to person, and differs from moment to moment (in the same site, same person). Living skin is very far apart from a mere gel-like structure, neatly layered (an assumption of many “layered electrical models of the skin”; see Section 1.3). Obtaining reliable and repeatable electrical measurements from humans frequently proves to be an exercise in frustration (the subject moves, breathes, perspires, vasoconstricts, or vasodilates; blood flows through the capillaries, etc., despite all attempts to “hold still for the measurement”). Searching the literature, engineers are usually confronted with medical terminology like “neurovegetative reaction”, “emotional state”, “rash”, and “placebo effect” that seem to be so gracefully handled by medical doctors but are impossible to translate in precise, quantitative numerical values. Often, some subtle medical phenomena are therefore left ignored by engineers.

Physical modeling of a natural process has many useful and practical applications, but for simplicity most modeling assumes a passive state of the observed process. For instance, measuring the electrical voltage drop at the ends of a wire allows us to model the wire resistance to the flow of current; the model might contain temperature, resistivity of the material, geometry of the wire, etc., and repeated measurements are similar (with sources of error that can be modeled, like thermodynamic noise, ambient electrical fields, etc.) However, living organisms have an additional key difference: they react differently based on the exposure to past events (including their past life experiences and past measurements). For instance, the act of measurement of human reaction time to a stimulus leads to an improvement in reaction time in subsequent measurements; this is true for many physiological characteristics where the human nervous system is involved [55]. A similar non-linear behavior was recently observed in skin electrical measurements: the result of a measurement is influenced by the previous act of measurement, prompting the characterization of the skin as a memristor [56,57,58]. Another limitation is due to the biases arising from the interaction between the human participant (subject) and the investigator, which can lead to erroneous recording or interpretation of the impedance data (discussed below).

## 2. Confounding Factors in Bioelectrical Impedance Measurements at the Skin Interface

### 2.1. Overall Physiological and Psychological Condition of the Participants

#### 2.1.1. Health Status of Participants

Systemic and/or local diseases can influence the properties of the skin; it is documented that diseases like psoriasis increase skin impedance, while atopic dermatitis lowers it [3,59,60], etc. But numerous other metabolic, endocrine renal, cardiovascular, and neurological diseases can profoundly alter the physiology of the skin and impedance recordings [61].

Recommendation: Even if the electrodes are not placed on (nor nearby) a skin lesion, the overall health status and relevant medical history of all participants should be clearly documented.

#### 2.1.2. Relevant Medical History

Medical history should be recorded via a written form, even for otherwise currently healthy participants. Past surgeries (for instance of the thorax, spine) can leave internal scar tissue or change local anatomy, therefore invalidating many assumptions of EIT analysis. A screening should be conducted for prosthesis of any kind, implants (active or passive), orthopedic screws, osteosynthesis plates, etc., as these have very different conductive or dielectric characteristics that alter BIA analysis [62,63]. These materials are not included in the BIA predictive models [64] and presumably also alter EIS and EIT accuracy. Current and past smoking status should be recorded; it is particularly important in EIT, as heavy smoking alters airway elasticity and thickness [65] and changes the recorded ventilation patterns [66].

#### 2.1.3. Core Temperature

Body core temperature and skin temperature are known factors that directly influence both the raw impedance values collected [61] and subsequent interpretation of the results [67]. Core temperature should always be recorded, and skin temperature should be in steady equilibrium with the environment. See Section 2.2.1 and Section 2.2.4 for additional details.

#### 2.1.4. Body Mass Index (BMI)

Particular attention should be paid to otherwise healthy overweight or obese participants. Elevated BMI changes skin physiology, stratum corneum (SC) thickness, and sebaceous and sweat glands; changes blood and lymphatic vessels; and changes transepidermal water loss [68,69]; each one of these modifications could alter the skin impedance measurements. Many BIA/EIS models assume certain limits for body parameters (obtained through statistical averaging on population); the body changes induced by severe obesity could be outside these limits and therefore alter the results of the modeling [70].

BMI of the participants should be recorded; during the data analysis phase, the BMI should be formally checked as a potential explanation variable in the statistical analysis.

#### 2.1.5. Age

From a physical point of view, the skin is a complex, heterogeneous, and anisotropic structure, and its response depends on many physiological factors. Age is one of the most important factors that increases large-scale anisotropy of the skin [71] and influences the skin impedance values [61,67]; SC moisture heavily depends on age [72]. Recommendation: the age of the participants should always be considered in the statistical modeling of the results, either as a continuous variable or as categorical (at least as “younger skin” vs. “elderly skin” [73,74]).

#### 2.1.6. Sex

SC moisture (and therefore the impedance) depends on the sex of the participants [72]. In EIT, the estimation of the total lung volume depends on various anthropometric variables, including sex [75,76]. We recommend that in comparative research paradigms to either do intra-group comparison or adjust for age and sex in modeling [61,67] as the biophysical properties of the skin differ [73].

#### 2.1.7. Menstrual Cycle

For precise inter-participant comparisons, phase of menstrual cycle (or menopausal status) should be recorded, as it has been found that some electrical impedance measurements are influenced by these factors [77].

#### 2.1.8. Ethnicity

There are contradictory or inconclusive data about the ethnicity relationship with resistance, capacitance, conductance, and impedance of the skin structures [78], while other studies have shown differences in signaling pathways and proteomics, which might explain differences in skin reactivity [79]. It is recommended to record it for the inter-participant modeling or adjustment [77].

#### 2.1.9. General Hydration Status

Skin hydration is one of the key influencers of the skin impedance via changes in local ionic concentration [67,80,81]. Perhaps a good practice would be to include explicit requirements for hydration in the experimental protocol, adapted to local climatological conditions (i.e., a statement like this: “participants in the study were required to maintain a normal level of hydration in the day, ingesting at least 2 L of water/fluids/day”, etc.). See also Section 2.2.2 for the closely related “Relative humidity” confounding factor.

#### 2.1.10. Feeding (Prandial) Status

The human autonomous nervous system changes state postprandially; there is a physiological phenomenon known as “gustative sweating” (sweat secretion that is induced by food ingestion) [82]. Therefore, it could be useful to add in the research protocol a condition like: “Participants abstained from food two hours prior to the investigation” (or other time amount deemed appropriate for the research question). For full body BIA measurements, many authors recommend fasting in the night before EIS measurement [61,67].

#### 2.1.11. Medication or Substance Use

A useful addition to the protocol would be the explicit exclusion of the participants that, for a given number of days before the measurement, were under medication or used recreational drugs. The rationale is that some of these substances could act on nervous, vascular, or endocrine systems, modifying natural skin responses. The researchers should judge if the use of coffee, tea, alcohol, etc., is acceptable or not and for which interval before measurements [61,67]; of course, this should be adjusted to the research question addressed.

#### 2.1.12. Sleeping Status

The participants should be adequately rested, as it is known that sleep deprivation changes sweating pattern and skin conductance reactivity to stress [83]. The alteration of sweating patterns in sleep-deprived participants appears to be broadly distributed on the surface of the skin and centrally controlled by the nervous system [84]. At a minimum, we could recommend screening for sleeplessness in the night(s) before the measurements, or record the number of sleeping hours in the previous night(s).

#### 2.1.13. Fitness

Body composition (distribution of lean muscle mass, fat, extracellular water, intracellular water) is different between athletes and non-athletes and can alter the predictive results from modeling measured impedance parameters (usually tend to underestimate body fluids in athletes) [85]. A recommendation would be to record the fitness status of each participant (athlete/non-athlete) to allow for adjusting in modeling.

#### 2.1.14. Exercise

It is known that high intensity training sessions or competitions temporarily change many physiological, biochemical parameters [86] and bioimpedance recordings [87]. A sensible recommendation could be to instruct participants to refrain from high intensity effort training 48 to 72 h before the EIS/EIT measurements and to refrain from any effort at least one hour before measurement (and rest) [67].

#### 2.1.15. Emotional Status

The psychological well-being should be checked, at minimum with the requirement that prior to the measurements the participants should not be exposed to stressful situations (via a short interview or checkbox–type form). It is known that emotional sweating is a physical reaction to emotive stimuli like stress or anxiety [82]; sweating modifies the skin impedance. Particularly concerning could be a physiologically healthy participant but with a generalized anxiety disorder due to prolonged or intense stress in previous weeks. We could recommend that, at least prior to the first measurement, the participants should be surveyed with a quick screening questionnaire such as Generalized Anxiety Disorder—7 Item Scale (GAD-7) [88].

As a concluding remark for this section, in our opinion these fifteen overall physiological confounding factors should be carefully accounted for in bioimpedance measurements involving electrodes on the skin, as these factors subtly alter the physiology of the sweat glands and therefore the moisture of the skin and of the skin–electrode interface. The functional status of the sweat glands appears to influence electro-osmotic transport of water in the skin patch beneath the electrode [89]; this correlates with the observation that the non-linear electrical properties of the skin have both rapid and slow variations in time [90]. The non-linear behavior, similar to a memristor (memory resistor), appears to be driven mainly by sweat ducts and sweat duct interactions with the surrounding cells and stratum corneum, with recent researchers proposing labels such as “sweat duct memristor” and “skin memristor” to describe their behavior [56,91].

Studies with human participants should be ethically conducted in accordance with the Declaration of Helsinki of the World Medical Association [92,93] and approved by the institutional ethics committee (or the ethics review board); written informed consent must be obtained from the participants. Researchers should exercise due care while recording and storing the screening data (anatomical and physiological) of the participants. Anonymization measures must be followed while processing and storing personal identifiable data; also, the bioelectrical signals must be treated as personally identifiable data, as many of them have unique biometric features among individuals [94].

### 2.2. Environmental Conditions

#### 2.2.1. Ambient Temperature

Ambient temperature directly influences the skin impedance and electrode–skin impedance measurements [95,96]. The main mechanism appears to be thermodynamic in nature: increase of temperature decreases resistance via enhanced ion mobility, determined in vitro [97].

Additionally, in vivo, hotter environments tend to create a reflex vasodilation in the skin, increasing local blood flow and presumably the conductance [53]. At the same time, perspiration also increases, and opening of the sweat ducts is one of the main contributors to reduction in impedance. Humans have a highly variable number of sweat glands (~20 to 700 per square centimeter); there is a sizable amount of variance on the skin of the same individual [82].

Colder environments do the opposite; in addition, colder environments can trigger a pilomotor reflex (“goose bumps”) of the arrector pili muscles (which can interfere with the placement of the electrodes and/or with the recording of electrical bio-potentials. Additionally, the contraction of the arrector pili muscles appear to squeeze out the sebum from the sebaceous glands [41]; the excess sebum might create an additional interface at the skin–electrode level, thus changing the measured impedance.

Based on ion dynamics, some authors therefore recommend trying to achieve as high as possible (and comfortable) skin surface temperature, even with the help of heated electrodes [95].

Other authors found that pre-warming the skin in warm water reduces skin impedance by 50% (measured with surface electrodes on dry skin) [53].

However, these methods of warming skin locally or generally have the risk of creating a sweating reflex and creating an error-inducing sweat layer. This is particularly concerning in areas rich in sweat glands, such as palms, feet, or the forehead. Careful investigation of this phenomenon led to the conclusion that “even imperceptible palmar sweating may equalize skin impedance over the entire palm” [43]. This equalization phenomenon also nullifies the notion of a control location if, for instance, the study investigates two adjacent skin regions for detecting differences in impedance between them [43,98].

We would like to re-emphasize the fact that probably the most understated factor in electrical measurements of the skin is the *living* sweat duct, which can sometimes suddenly act like an electrical short-circuit [99] or as a complex electrical element (memristor) [56].

#### 2.2.2. Ambient Relative Humidity

Exposure to dry air increases the evaporation rate and desiccation of the outer layers of the skin (leading to an increased impedance). Exposure to humid air will do the opposite; excessive humidity can create condensation effects on the surface of the electrodes or equipment and might lead to spurious readings. The relative air humidity was found to directly influence the skin impedance [96,100]. The relative air humidity, together with general hydration status, are the main drivers of the skin hydration level (see Section 2.1.9).

#### 2.2.3. Stability of Ambient Conditions

An air draft can also trigger the pilomotor reflex in some individuals; this can also happen during rapid changes in the ambient temperature (for instance, produced by a swinging jet of an air conditioner or a fan).

Radiative heat sources too close to the participant (heaters or direct sunlight from a window) can directly heat skin layers and induce perspiration and vasodilation (see above).

Recommendation: Ambient temperature and relative humidity should be recorded and reported in the study. These parameters should be kept constant during a recording session and ideally identical for all sessions; this can be achieved reasonably easily with a good automatic climate control system.

It should be verified that during the whole EIS/EIT procedure, there are stable, similar conditions for all participants. The participants should not be seated in places where there is risk of sudden flow of air-drafts, or in direct radiative heat emitted from heaters or sunlight.

Few EIS/EIT studies report both temperature and relative humidity and also the steps taken to maintain them as constant for inter-participant comparisons; these are important indirect confounding factors in impedance measurements through their influence of the skin water content (via evaporation, perspiration).

#### 2.2.4. Accommodation to Laboratory Conditions

Since skin surface temperature is a known error factor for modeling parameters in BIA [67], the research protocol should include an explicit time period (for instance, 15 min) to allow accommodation of the participant to the laboratory environmental conditions, and to let the skin surface to reach a thermal steady-state relative to the surrounding air. As a practical recommendation, this waiting time can be used to fill in consent statements, screening forms, etc.

#### 2.2.5. Posture and Postural Accommodation

Additionally, during this time, the participants should also ideally be seated in the measurement bed/chair (depending on the EIS, EIT, etc., performed) and minimize their movement as it is known that postural changes affect the relative distribution of extracellular and intracellular water, with different time constants (5 to 30 min) [101], which can induce unwanted variability in bioimpedance analysis [61,67,102,103].

Posture during the experimental recordings should be standardized for all participants, regardless of the method, as it is known that posture influences whole body EIS and EIT [76,104].

#### 2.2.6. Season

The impedance of the same skin area changes systematically with season, with quantifiable differences between summer and winter [105]; this seems to be related to the relative change in SC thickness changes with skin exposure to UV solar radiation, or changes in molecular components [106]. Additionally, SC moisture content depends on the season [72]. A practical recommendation, especially for investigative research studies, would therefore be to schedule the measurements in a single season for all human participants, or to adjust for seasonality in the case of long-term studies.

### 2.3. Time Constraints

#### 2.3.1. Time of Day

Circadian rhythms influence many physiological or neurological phenomena, and there are measurable changes at the biochemical level in many tissues [107], so the time of day might be a confounding factor for EIT/EIS measurements. Measured skin dielectric constants appear to vary slightly but significantly during the day (from morning to evening) [108,109]. These modifications appear to be related to the significant diurnal variations in skin thickness due to normal dermal fluid translocation [110].

We suggest that the time of day could also be considered a confounding factor, and that measurements should be ideally performed at the same hour of the day for all participants.

#### 2.3.2. Timing of the Measurement: Immediate or Delayed After Electrode Placement

Some studies reported the beginning of the measurements immediately after the placement of the electrodes. Experimental and theoretical studies have shown that skin impedance “drifts” (changes in time) from the beginning of the measurements. A drift of only 5% from the starting impedance is serious enough to cause significant recording errors (especially for EIT), and some authors recommend at least 10–15 min delay between the placement of the electrodes and the recording to allow the electrode–skin interface to stabilize [37,111].

Depending on the electrode type, the research protocol should include a timed delay between the electrode placement and the data acquisition.

#### 2.3.3. Measurement Repetitions in Time

It is known from earlier measurements that the impedance of the skin (regardless of the site or other factors) increases significantly with repeated measurements, regardless of device type [43]. The more measurements are performed in a shorter time frame, the higher the effect. The research protocol employed should include a fixed number of repetitions in a given time frame (and in cases of failed measurements, allow a recovery time).

### 2.4. Skin Condition

#### 2.4.1. Tattoos, Piercings

Metallic, conductive, or dielectric additions to the skin could modify the skin impedance readings. For metallic objects (piercings) inserted in or beneath the skin, it is obvious that they can profoundly alter the path of electrical currents and the shape of the electrical fields. A less obvious confounding factor are tattoos. Some modern tattoos use inks with electro-conductive residues (iron, nickel, chromium) and can create highly conductive patterns in the skin [112].

We recommend screening the participants for the presence or absence of tattoos in any location of the skin, as these can be a confounding factor. We stress that even if the conductive tattoo or piercing is farther from the investigated location, a high resolution EIS or EIT can theoretically be influenced by their presence. If screening is not possible, at least record their presence (in a yes/no variable) so this information can be used in modeling.

#### 2.4.2. Items in Contact with Skin: Jewelry and Conductive Clothing Accessories

Care should also be taken to the presence of electro-conductive items like jewelry (rings, necklaces, bracelets, watches, etc.) [61,67] or parts of clothing (buckle clasps, metallic buttons, etc., in contact with the skin) that can act like current sinks. We recommend that these should be removed during the impedance measurements.

#### 2.4.3. Items in Contact with Skin: Clothing, Belts, etc.

The main issue is tribo-electric static charge generation. Clothing made from natural cellulose-based fibers (cotton, linen) is less susceptible to dielectric polarization (like synthetic fibers—polyesters, lycra, etc.) [113]. The build-up of static electricity is dependent on the materials, weave patterns [114], layering, and movement amplitude [115,116]. We recommend that the participants should wear comfortable clothing, ideally made from cotton or linen, with as few layers as possible (to minimize the friction area). To further reduce the chance of spurious electrostatic discharges from clothing, it could be a good idea to earth the garments via a bonding clip and a 10 MΩ resistance to earth before the impedance measurement [117], then minimize movements of the subject until the impedance measurement is complete.

We suggest to pay attention to the interaction between the clothing and the textile covering of the bed or chair where the measurement is performed: to minimize static build-ups, the bed or chair can be lightly wiped with a dryer sheet with antistatic agents, then covered with a single-use cover-sheet made from cellulose-based paper. The chair or bed frame can be also be earthed with a high-resistance wire prior to measurements.

Clothing should be dry (not moist nor wet due to perspiration). This condition should be thoroughly checked before and after the measurement, as sometimes we notice that after prolonged sitting on medical beds with an insulating surface, the local temperature of the skin increases, and there could be a focal sweating on the contact spots on the clothing of the patients. The same observations apply for other items that are in contact with the skin or in proximity to the electrodes (for instance, belts or bandages supporting the electrodes).

#### 2.4.4. Local Health of the Skin

Before any impedance measurements, it is worthwhile to re-check any spurious intervening event that could affect a local patch of the skin, like insect bites, bruises, cuts, rashes, scratches, pressure zones, ingrown hairs, previous small wounds from medical procedures like injections, etc.

The cells in the layers of the skin have different ionic concentrations in their cytoplasms. In normal healthy skin this creates a vertical transepidermal potential of about 15–60 mV (inside positive; absolute values varying with location on the surface of the body) [45,118]. In a wounded skin, this generates an injury current; the electrical fields on the sides of a wound can reach 40 mV/mm. This seems intense enough to stimulate keratinocytes’ migration in the wounded area (to begin re-epithelization) [118]. This creates some challenges in recording EIS/EIT in wounded skin. For instance, it is documented that small puncture wounds from needles modify skin impedance recordings [119].

In our opinion, the research protocols should include an explicit check of small accidents that the participant might not be aware of (like small bruises, cuts, etc.) or minor medical procedures close to the electrode sites. These alter the skin integrity and the relative thickness of its layers, adding another difficulty to the intra- and inter-subject comparisons.

### 2.5. Skin Preparation

#### 2.5.1. Topical Hygiene Products

Particular attention should be paid to the habitual usage of various cosmetic or hygienic products like moisturizing creams, moisturizing shower gels, moisturizing shampoos, etc., as they can alter the skin impedance [120], significantly altering the impedance modeling [121]. The main effects appear to be due to the content of salts of organic acids and/or emulsified lipids in skin moisturizer products. The effect of moisturizers on SC impedance appear to be erratic, with large differences between products and between different individuals [122]. Their effects on impedance seem to wear off completely after one to four days after application [122]. Some components of regular cosmetic products are also “penetration enhancers” (see Section 2.5.3) that increase skin permeability (and can reduce impedance). Other components can actively block the sweat glands (various antiperspirants).

Therefore, a protocol recommendation could be to instruct the participants to avoid (or use in a controlled form) any skin moisturizer or other cosmetic product application (in particular, topical creams or lotions), at least for four days before the EIS/EIT measurement day.

#### 2.5.2. Skin Hygiene

Over time, the skin surface accumulates skin oils, sweat, dust, or cosmetic products; these should be removed prior to measurements, as these would be unknown variables in the skin–electrode interface. A standardized procedure should be established and followed consistently for the preparation of skin beneath the electrodes, as this is crucial for a good, stable contact with the electrode. It is essential that all the participants follow the same procedure, but this is easier said than done. There are different methods used, each one with pros and cons:

- Water-and-soap: effective, but has several downsides. Harsh surfactants can cause skin dryness and irritation mainly due to damage to skin proteins [123]. As a recommendation, less irritating, “mild” surfactants exist, designed to alleviate this problem [124,125]. Additional care should be paid to the pH of the soap mixtures with water, as strong alkaline ones (pH ~ 10) increase SC thickness (by swelling and increasing lipid rigidity), even in the absence of surfactants [123]. Therefore, recommended soaps should have a neutral pH (~7) or close to the pH of SC (~5.5). Some soaps also have moisturizing agents (see above). Another downside is that it exposes the skin to water, which transiently increases the water content of the superficial layer (increases conductivity) or might leave a thin water film on the surface. Tap water might contain variable levels of oxidizers or reduced residues from water management (ozone, chlorine, potassium permanganate, polyphosphates, etc.) that might alter redox equilibrium at the electrode interface [126]. Countermeasures: (i) rinse effectively (maybe with normal saline instead of water); (ii) dry effectively (and include a timed interval between the skin preparation and electrode attachment).
- Rubbing alcohol (either ethanol or isopropyl alcohol in various concentrations): It cannot effectively remove proteinaceous residues that might clog sweat ducts, as proteins denature in concentrated alcohol solutions. Also, it removes skin oils in the hair follicles, which will slightly reduce the impedance temporarily [4] and might cause irritations. Therefore, cleaning with alcohol should be avoided if possible.
- Gel-based hygienic agents: These off-the-shelf products can contain various thickening agents that can attach to the SC as a thin additional layer; we recommend avoiding them.
- Single use cleaning wipes: These can be effective but check for the presence of moisturizing agents and pH; abrasive wipes are commercially available for slightly abrading the SC.
- Sterile woven cotton gauze (surgical cotton cloth) moistened in normal saline. When rubbed against the skin, this appears to be a safe option that we recommend, with the following observations. The texture appears to be effective for gentle, non-traumatic exfoliation, and the absorbent fibers of the cotton mop up oils and debris. We advise using normal saline (sterile 0.9% sodium chloride solution) and not water to further minimize osmotic water flux through microscopic cracks in the skin. The number of rubs per electrode location should be standardized in the research protocol (i.e., “each location was rubbed four times before with a woven cotton gauze moistened in normal saline”). For each electrode location, use a new cotton pad (to prevent cross-transfer of debris between locations).

#### 2.5.3. Stratum Corneum: Modifying or Not

The influence of the very variable thickness and characteristics of the SC pose methodological challenges in recording and modeling. Some authors propose to avoid SC altogether by stripping it; this was done even from the earlier studies. Skin impedance is greatly reduced if the surface layer of the skin is abraded [4].

- For stripping, the most used method seems to be the use of adhesive tape (taped to the skin, lifted rapidly), several times (one to four times most common). Some authors specify the type of tape used (generic plastic based/cellulose based), others do not; also, there are commercially available tapes specifically marketed for the purpose of stripping the SC. Other authors used other stripping methods (for instance, fine-grained sandpaper rubbed on the skin). Mechanical abrasion seems to be the most effective method to reduce impedance [127].
- Other authors prefer not to use stripping, for the following reasons: because it is unpleasant, cannot be applied on hairy skin easily, reduces participation, modifies the natural state of the skin, severe stripping (~20 times) denudes completely the skin protection and increases the risk of infections. Accordingly, lack of stripping will induce a variability between subjects. We want to note that even if the study authors did not use stripping, it might be done inadvertently by the participants, if they, in their hygienic habits at home, used cosmetic exfoliating soaps or gels or hard sponges or brushes (that can have a stripping effect). If the choice of the researcher is to avoid stripping, the participants should perhaps be instructed to refrain from using exfoliating cosmetics for the duration of the study (especially if the study design has repeated measures in time). This could be an easy to miss confounding factor in longer duration studies.
- Another mechanical abrasion recommended by some researchers involves the use of an abrasive electrolytic gel, rubbed on the skin [95]; the application should be carefully performed, not to spill the gel into adjacent areas (it could create an electrical shorting between neighboring electrodes).
- Other modifications could be chemical exfoliation with different agents or microperforation with microneedles [127], but these appear to be less effective than stripping.
- “Penetration enhancers” are substances that increase the permeability of the skin for certain species. Surfactants (like sodium lauryl sulfate, commonly used in shampoos, soaps, etc), solvents (like dimethylsulphoxide), esters of organic acids, and aromatic compounds seem effective in reducing the parallel resistance of SC [95], but their use is severely restricted by their toxicity, or risks of irritations and allergies.

It should be noted that SC modification seems to be unsuitable for longer duration studies, where the electrodes are supposed to stay continuously attached to the body, as the skin regenerates SC in approximately 24 h [127].

#### 2.5.4. Shaving

The contact surface between the electrode and skin is affected by the normal hair follicles; the contact surface area is thus variable and leads to spurious changes in the impedance readings. The presence of hair significantly changes the impedance readings [128]. Due to friction, hairs can pull on the underlying skin, possibly leading to motion artifacts [129]. If the chosen location for the electrode placement is in an area with hair follicles, a decision should be made about shaving. Wet shaving is effective but has the downside of hydrating the skin, and sometimes the aftershave lotions can have oils or moisturizers that can linger on the skin (see Section 2.5.1). These should be avoided and skin dried effectively. Dry-shaving can mechanically induce vasodilation and irritation.

From the electrical point of view, a shaved skin area below the electrode seems to reduce variability of the contact area interface. Because of the above-mentioned problems, shaving should probably be done at least several hours before the recording or on the previous day. If a “must shave” decision is taken, care should be taken about the inter-subject comparison: is the procedure being performed in all participants (as there is a large difference in hair distribution)? Or perhaps an additional adjusting variable should be added to the analysis to perform a finer control.

For these practical reasons, some authors prefer to investigate points on the body parts without hair follicles (palms, soles, forehead). Unfortunately in these regions there is a higher distribution of sweat glands [82], which add peculiar unexpected difficulties in controlling the measurements (see Section 2.2.1). Vexing as it is, focusing the measurements only on the areas without hair (in order to reduce variability of the electrode contacts), could actually be a cause for a huge variability of the impedance readings due to the higher number of sweat ducts in these areas.

### 2.6. Electrodes

A complicated choice has to be made about the electrodes; there is a huge amount of research devoted to electrodes that is outside the scope of this review. We will highlight here only the main problems that can arise for each type of electrode. Briefly, there are two main sources of electrodes: (i) some researchers are using stock electrodes that are produced by third parties or produced by the manufacturers of the measurement equipment; (ii) other researchers are using custom-made electrodes for their own research purpose (for novel biosensors or for less researched diseases, etc.). Depending on how they make contact with the skin, electrodes can be classified in various ways, but for the scope of this review we categorize them as (1) “wet”, (2) “dry”, (3) “inserted”, and (4) “non-contact” electrodes. Extensive reviews are available discussing the electrode types, used in various research paradigms; for instance, [7,80,99,130,131,132,133]. Each type of electrode has its own advantages and challenges, and we will highlight here only the skin-interaction problems associated with each category.

#### 2.6.1. “Wet” Electrodes

They are characterized by the existence of a liquid contact layer between the skin and the rest of the electrode [95]. The main materials of the wet electrodes are as follows: (i) the “gold standard” in bioelectric measurements is the silver/silver chloride (Ag/AgCl) electrode due to its stability. Many variations exist, with various conductive gels between Ag/AgCl and the skin; (ii) various conductive gels between a solid metallic electrode and the skin [134]; (iii) cotton or other textile swabs with conductive saline solutions (like potassium chloride, but also sodium chloride was used in the past); (iv) conductive saline solutions directly on the skin (maintained in place with rubber rings, cylinders, or suction cups).

- Advantages: The presence of the liquid/gel media increases the effective surface area by eliminating random tiny air pockets, resulting in a stabler impedance signal over time. The initial electrical contact is usually stable and reliable. The electrodes based on adhesive gels are very easy to apply and are used extensively in hospitals in electrocardiography (ECG) recordings; their ubiquity made them a popular choice for many impedance research projects.
- Disadvantages: The presence of a gel/conductive saline solution moistens the skin, thus the skin impedance is lowered. This process is not stable because over time the conductive liquid can infiltrate between the tiny cracks in the stratum corneum, making spurious conductive bridges with the underlying layer. Also, the outer layers of the gel can dry, modifying conductance; thus, the gel–skin interface can actually generate an unwanted noise over longer recording [80]. Most of these electrodes are single use; some participants might be allergic to a particular gel formulation.

#### 2.6.2. “Dry” and Other Contact Electrodes

These are various conductive items placed in direct contact with the skin [135,136,137]. Materials are diverse: (i) solid metal electrodes like stainless steel, titanium, gold; (ii) elastic/stretchable materials like electroconductive rubbers, elastomers; newer electroconductive textiles; skin-like bioelectronics made from various materials [138,139,140], etc.

- Advantages: Most types are designed to overcome the problems of wet electrodes (easy to use, reusable, comfortable) and can be used easily in any location of the body, but they come with their own challenges. Flexible contact electrodes have the advantage of high conformability to skin shape (even on hairy regions) and if they are elastic are able to maintain the interface even in motion (like breathing or exercising) [138]. In general, because of the lack of a contact liquid or gel, the absolute impedance values recorded are generally higher than those recorded with wet electrodes. Newer conformal electrodes appear to maintain stable electrical contact for longer durations in EIT applications [141].
- Disadvantages: Even if they are not “wet” in a chemical sense, an electrical double layer forms in time at the contact with the skin (from natural sweat/oils that the underlying skin might secrete during the usage). This results in a variable contact impedance that “drifts” over time from the initial value [136]. There are attempts to minimize this effect via porous mesh-like electrodes [142]. Dry contact electrodes (especially solid ones) are highly sensitive to motion artifacts [143]. The motion artifacts have a large variation between subjects and appear with overt motion and also with involuntary movements (like breathing) [22].

#### 2.6.3. “Non-Contact” Electrodes

These are not “electrodes” in the classical sense but are functionally equivalent; they are also known as “insulating electrodes” or “capacitive electrodes”. A small metal plate is placed above the skin (very close, but not in contact); the electrolyte (liquid or gel) in contact with the skin is replaced by a dielectric environment (an insulating polymer, etc). The plate is electrically charged, and an electrostatic field establishes between the skin and the plate (the ensemble works as a capacitor). Any tiny variation of the electrical properties of the underlying skin immediately changes the capacitance of the system and induces a minuscule current (~picoamperes) in the metallic plate. This is detected by a highly sensitive amplifier. There are many types of non-contact electrodes [144]. For instance, by using a non-contact electrode with a thin air gap as an insulator (a Kelvin probe), mounted on a scanning arm, a detailed 2D map of the electrical properties of skin were obtained [145] as an alternative procedure to mapping skin impedance with contact electrodes. The main disadvantages of this promising method are (i) that it requires dedicated, specialized equipment, and (ii) it is very susceptible to electromagnetic interference (see Section 2.8.2); therefore, it cannot be performed without special shielding (a Faraday cage enclosing the whole setup) and vibration dampeners, which are cumbersome to use properly in a clinical setting.

#### 2.6.4. “Inserted” Electrodes

These are needle-like electrodes, made from various materials (medical grade stainless steel, platinum, iridium, gold, etc.), inserted in the skin. For instance, electrodes used for electromyography (EMG) recordings can be used unmodified or be specially adapted for four-electrode method impedance measurements [146]. Most needle electrodes have one or more small conductive regions (usually around the tip) and an insulation layer (usually Teflon).

- Advantages: They avoid the variability of the contact between the applied electrode and the skin surface, discussed above. They appear to have a better signal-to-noise ratio than the rest of the electrodes [147].
- Disadvantages: (i) The geometrical arrangement of the needle electrode surface influences the recordings: at lower frequencies of the electrical signal, they have a high, significant electrode polarization impedance; the impedance of the surrounding tissue was thus best recorded at higher frequencies above 10 kHz [148]. (ii) As an additional source of error, most of these electrodes have to be “activated” before measurement with different procedures (typically by dipping them in saline solution with wetting agents or on-site electrolytical treatments). This allows a stabilization of the electrode surface area, reducing the variation of electrode impedance observed during the in vivo recording. This phase must be followed carefully before the measurement; the results (from the same electrode) are markedly different with or without this pre-treatment [148]. (iii) Needle insertion is a medical procedure that breaks the integrity of the skin barrier, and proper sanitary precautions must be followed to avoid infections or medical complications. It might be painful and less likely to be accepted by the participants/patients than alternatives. A promising solution to these problems appears to be “microneedles” arranged in an array that can penetrate the nonconducting stratum corneum of the skin without pain and can even be arranged in multichannel setups [149].

#### 2.6.5. Electrode Surface Area Considerations

A significant part of the noise component of the bioelectrical impedance measurement originates from the electrode–skin interface; the magnitude of the noise appears to be inversely proportional to the square root of the area of the electrode on the skin [150]. The variability of the electrode–skin interface appears to be more important than the local spatial anisotropy of the impedance of the skin [43]. It seems reasonable then to use smaller diameter electrodes (1–2 mm) instead of larger ones; unfortunately, this gives rise to another problem: local current density beneath the electrodes.

- “Small” electrode area. Advantage: It leads to higher density current lines below the electrode, which has a higher chance to intercept the interest area (for instance, an unknown low impedance anatomical structure that was to be investigated in an impedance spectroscopy study or impedance tomography study). Disadvantage: Higher current density means a higher chance for the sudden appearance of burned-through low impedance paths in the epidermal layer (and thus a false low impedance reading) [99].
- “Larger” electrode area. Advantage: It is easier to use and attach. Lower current density beneath the electrode reduces the chances that the tissue is adversely affected by the measurement, but also lowers the signal-to-noise ratio. Also, a larger perimeter of the electrode is a source of other confounding factors (noise at the electrode–skin interface [150] and, especially in the case of wet electrodes, a greater surface area of evaporation, so the gel will desiccate faster).
  Newer generations of “compound electrodes” [151,152] that have a large outer electrode area to inject current and a smaller inner area to sense voltage are actively designed to work around the above described problems. “Active electrodes” are a newer generation of electrodes that incorporate parts of the controlling electronics for better signal-to-noise ratio [153,154].

Conclusion of this section: There are numerous types of electrodes, but none was found to be stable over time for the various reasons outlined above. Sadly, there is no “best electrode” yet and there is ongoing research to develop newer electrodes (especially for wearable devices). From a practical perspective, for a given impedance research study (EIS/EIT of a given structure) it is often difficult to test all electrode types. Therefore, for the types of electrode available, researchers should judge if, for their research project, the advantages outweigh the disadvantages. The fact that all currently used electrodes are unstable over time should always be accounted for as a source of error in statistical modeling of the results.

Detailed comparisons of the relative performance metrics of various electrodes have been made, and it was found that, for the dry electrodes, the impedance changes in time are much larger than those of other electrode types [155,156]; despite the shortcomings, the dry electrodes still have the best usability [157], being easier and faster to apply. Improvement of the stability of the electrodes is being actively researched [158,159], with promising designs: (i) ultra-thin tattoo-like dry electrodes [156,160,161], able to maintain a dry interface with the skin [162]; (ii) mixed-fabric dry electrodes [163,164]; (iii) high-entropy alloys, with a greater thermodynamic stability and thus potential longer longevity of the electrodes [165,166]; or (iv) mixed ionic-electronic conductive ultrasoft materials [157,167,168]. Hopefully, these new directions will lead to better general electrodes in the near future.

### 2.7. Electrode Placement

#### 2.7.1. Pressure on Electrodes

Precise measurements have shown that pressure applied on the electrodes influences the impedance measurement [31,95,169,170]. Skin–electrode impedance decreases with applied pressure [136], perhaps due to increased effective contact area or to forceful elimination of small amounts of sweat in the deep glandular part of the sweat glands. Changes in skin–electrode impedance were prolonged, lasting even after the applied pressure was released [171]. However, as the pressure increases (from 1 kPa to 50 kPa), in moist areas, an opposite effect happens, perhaps due to the squeezing out of water from the intercellular space [172].

It is practically difficult to rigorously control the applied force; it can be done with the aid of force sensors, (striving to apply all electrodes with the same force, [43,173]). Since this can be impractical in most clinical situations, an investigation should be done about the error rates for the particular method employed. Other studies did not control for the pressure, so their results might inadvertently be influenced by this factor. See also Section 2.9 below for an additional discussion on pressure.

#### 2.7.2. Electrode Location

Careful electrode placement is of paramount importance for good impedance measurement. For some devices like full-body BIA analyzers, or other commercial devices, standardized positions of electrodes are indicated by the manufacturers. For research purposes, sometimes the investigators choose different positions. For each research question addressed by a study, an investigation of the most suitable electrode location should be performed, as the anatomical location of the electrodes is known to influence the results. For instance, the clinical usability and plausibility of EIT measurements depend on proper belt position; for monitoring lung volume changes, the best electrode plane seems to be between the 4th and 5th intercostal space [174].

The research protocol should include specific details about the precise location of electrodes (relative positions to anatomical landmarks). Care should be exercised and training provided for technicians or operators about dealing with common skin anisotropy problems that might be encountered in the chosen location (presence of skin scars; large benign nevi; large telangiectasias or other benign skin changes), as these might interfere with electrode placement. It is perhaps better to explicitly state an exclusion criterion in the research protocol for these situations.

### 2.8. General Problems Related to Hardware

Proper equipment maintenance, calibration, and set-up should be performed; connectors should be checked for oxidation and sound electrical contact, etc.; this is outside the scope of this review. We would like to highlight some problems related to the hardware–skin interface.

#### 2.8.1. Wires

An electrode is connected to the recording amplifier via a conductive wire (connector). Especially in the case of wet electrodes, over time the wires can be affected by internal corrosion: humidity and external substances can infiltrate inside the wire, through tiny cracks in the insulation layer. Internal corrosion increases the internal resistance of the wire, which can create erratic readings in impedance (depending on the wire movement). A very good practical advice, especially for four-point measurement systems, is given in [20] about a practical way to check the internal consistency of the connection between the electrodes and amplifier: keep intact the wire–electrode connection, but reverse the leads at the amplifier and remeasure the resistance. The two measured resistances should be within 10% of each other, otherwise there might be a problem with the setup.

#### 2.8.2. Equipment Location

A suitable, stable location should be chosen such as to minimize wire movements and avoid electromagnetic interference. AC power conduits in walls, poorly designed electronics of fluorescent or LED (light emitting diodes) light fixtures, UPS (uninterruptible power supply) units, microwave ovens, freezers, other medical equipment, etc., can create electromagnetic interference in the environment nearby.

### 2.9. Sources of Bias in Human Trials

There is a huge body of medical research dedicated to avoiding selection bias, performance bias, detection bias, attrition bias, reporting bias, and other biases [175,176] that frequently appear in human studies. Since the bioelectrical impedance studies we reviewed also deal with human interactions, different biases can arise. A decision about the mitigation strategies for each bias should be made in the protocol of the study. We highly recommend following already standardized guidelines for avoiding biases in clinical trials (and we reference as an example [176], with online tools for aiding). We would like to provide an example of a performance bias with a made-up example, to highlight the subtleties of human factors in electrical measurements of the skin:

For instance, in an EIS study looking to identify the skin impedance changes between a “reference group” (healthy participants) and a “condition group” (let us say, patients of a rare, debilitating disease). Wet electrodes are deemed unsuitable for the condition and an EIS study is designed around dry electrodes that have to be attached onto the arm with a fitted wrapping bandage and worn for 24 h. A performance bias can arise if the operator of the EIS device fits the wrapping as designed on the control group, but being emotionally involved, wraps loosely the electrodes on the skin in the condition group (“in order not to inadvertently discomfort the patients”). Therefore, the impedance results will be different due to the result of the unequal, uncontrolled pressure on the electrode.

There are effective strategies for reducing biases; the first step is recognizing and identifying them at the planning stage of the study. One way of identification is to represent the variables of the study on causal diagrams (directed acyclic graphs); see [177] for visual examples of graphs for the biases discussed above. A discussion of the investigation protocol with a researcher trained in human psychophysics or an epidemiologist could provide very valuable hints about particular sources of bias.

### 2.10. Data Modeling

The recorded data (the impedance values of the tissues) should ideally be modeled and not analyzed directly. For instance, for the skin impedance there are several standard models of the electrical behavior of the skin (Cole–Cole model, Montague, Tregear, Lykken, Constant Phase Element model, etc.). Each model has advantages and disadvantages, and the use of an equivalent circuit model is always limited by inherent errors [19,28,39].

Choosing to use a particular electrical model of the tissues, a researcher makes (aware or not) an approximation that may significantly affect the accuracy and validity of the subsequent interpretation of the data [39]. We highlight the fact that an equivalent circuit model is just a model (not the reality of the underlying structure). We suggest [39] as an in depth review of the basic mistakes, misunderstandings, misnomers, limitations, and erroneous interpretations that unfortunately cropped up in published research using impedance spectroscopy employed in tandem with equivalent circuit modeling. McAddams & Jossinet (ante cited, [39]) observed that, unfortunately, in some cases a considerable amount of good experimental work was wasted by using an inappropriate model. Further, they remind us that the modeling work is a difficult one, and it seems that sometimes the researchers used the model for which the appropriate software or hardware existed in the lab (and not the model that was more likely to be correct for the particular topic researched). For impedance spectroscopy, a wide frequency range must be used, and a large number of data-points must be collected in order to make a meaningful interpretation of the data.

Statistical data modeling poses another challenge. Simpler statistical modeling comparing groups by impedance values (linear regressions, t-Student tests, ANOVA) might be ineffective due to huge range variations and non-linear aspects of the phenomena. A serious problem is that, most of the time, the data are “noisy” and the classical statistical methods are not sensitive enough for picking up differences. Better approaches to statistically compare noisy data are principal component analysis (PCA) [178,179,180,181], linear mixed-effect modeling (LME) [182], and general additive modeling/general linear mixed models (GAM/GLMM) [183].

## 3. Conclusions

We have identified forty possible confounding factors that might alter the accuracy of impedance measurements at the skin interface level. These involve medical aspects (physiological, anatomical, pathological), electrical phenomena (electrodes, electrical methods, instrumentation, etc), the environment (external factors that could influence the setup), and data interpretation (modeling and analysis), as well as psychological factors (anxiety disorders, neurovegetative reactions, operator bias, etc.). It is important to note that some factors have significant interdependencies and overlapping (e.g., ambient humidity and hydration status); these interdependencies increase the risks of confounding if each factor is not individually accounted for in the study design.

In our opinion, this review can be used as a checklist for future researchers wishing to reduce as many confounding factors as possible in their own research. We propose that certain steps taken before and during the acquisition of data might reduce error rates. At a first glance, the error rate for each step seems to be small, but if unaccounted for the errors might sum up to a level that can drown the useful signal in noise.

A limitation of this study is that, unfortunately, it has not been possible to provide a quantifiable amount of the relative influence on error rates of all the factors. There is a consensus that there is a need to reduce error rates via standardization [61,184], especially in diverse demographics [185]; still, the reconstruction algorithms cannot be routinely applied to ill patients with physiological parameters that largely deviate from the population mean (e.g., extreme BMI or severe electrolyte imbalances) [186]. This limitation was due to the paucity of studies of the specific relationships between confounding factors and highlights the need for further research in this area.

A “perfect experiment” that completely eliminates measurement errors is out of reach [187,188], but identifying and reporting as many error factors as possible is crucial, especially for clinical research [189]. While planning for an experimental measurement, a researcher could find the summary in Table 1 useful. Next to each factor, the researcher could mark whether the confounding factor was fully mitigated (Yes), was not mitigated (No), was only partially mitigated (Partially), or does not apply for the current experiment (N/A). For instance, an “N/A” could be used for the menstrual phase if all the participants are men. Or, for instance a “Partially” could be used if in the cohort some participants were willing to participate but unwilling to remove a piercing in a distant location of the body.

In laboratory medicine, the “pre-analytical phase” refers to all requirements and procedures needed before the start of laboratory testing. This phase of the testing process is responsible for the majority of errors in laboratory medicine [190,191]. We advocate that a similar approach (accounting for variables such as age, sex, fitness level, BMI, feeding status, etc., performing certain standardized steps before each procedure) might also be beneficial for increasing the quality of electrical impedance measurements in medical applications.

We stress that the purpose of this paper is to aid researchers wishing to apply impedance analysis to their research paradigm. If a researcher uses a commercially available ready-made, approved device, some information presented might not be applicable (depending on the design of the electrodes, design, and calibration of device, etc.). Judgment and care should be employed, and a discussion of the issue with the designers of the device is a must.

## Figures and Tables

**Table 1:** Checklist for the mitigation of confounding factors in the experimental setup of bioelectrical impedance measurements.

Confounding Factor	Accounted for and Mitigated in the Experimental Design?			
	Yes	No	Partially	N/A
**Overall physiological and psychological condition of the participants**				
Health status of participants	□	□	□	□
Relevant medical history	□	□	□	□
Core temperature	□	□	□	□
Body mass index (BMI)	□	□	□	□
Age	□	□	□	□
Sex	□	□	□	□
Menstrual cycle	□	□	□	□
Ethnicity	□	□	□	□
General hydration status	□	□	□	□
Feeding (prandial) status	□	□	□	□
Medication or substance use	□	□	□	□
Sleeping status	□	□	□	□
Fitness	□	□	□	□
Exercise	□	□	□	□
Emotional status	□	□	□	□
**Environmental conditions**				
Ambient temperature	□	□	□	□
Ambient relative humidity	□	□	□	□
Stability of ambient conditions	□	□	□	□
Accommodation to laboratory conditions	□	□	□	□
Posture and Postural accommodation	□	□	□	□
Season	□	□	□	□
**Time constraints**				
Time of day	□	□	□	□
Timing of the measurement: immediate or delayed after electrode placement	□	□	□	□
Measurement repetitions in time	□	□	□	□
**Skin condition**				
Tattoos, piercings	□	□	□	□
Items in contact with skin: jewelry and conductive clothing accessories	□	□	□	□
Items in contact with skin: clothing, belts, etc.	□	□	□	□
Local health of the skin	□	□	□	□
**Skin preparation**				
Topical hygiene products	□	□	□	□
Skin hygiene	□	□	□	□
Stratum corneum: modifying or not	□	□	□	□
Shaving	□	□	□	□
**Electrodes**				
Electrode type	□	□	□	□
Electrode surface area considerations	□	□	□	□
**Electrode placement**				
Pressure on electrodes	□	□	□	□
Electrode location	□	□	□	□
**General problems related to hardware**				
Wires	□	□	□	□
Equipment location	□	□	□	□
**Sources of bias in human trials**				
Check for potential biases	□	□	□	□
**Data modeling**				
Check for appropriate modeling	□	□	□	□

## Data Availability

Not applicable.

## Abbreviations

The following abbreviations are used in this manuscript:

AC	Alternating Current
BIA	Bioelectric Impedance Analysis
BMI	Body Mass Index
EIS	Electric Impedance Spectroscopy
EIT	Electric Impedance Tomography
GAM	General Additive Modeling
GLMM	General Linear Mixed Models
LED	Light Emitting Diodes
PCA	Principal Component Analysis
SC	Stratum Corneum
UPS	Uninterruptible Power Supply
